# Gene and Cell-Based Therapies for Parkinson’s Disease: Where Are We?

**DOI:** 10.1007/s13311-020-00940-4

**Published:** 2020-10-30

**Authors:** Philip C. Buttery, Roger A. Barker

**Affiliations:** 1 Cambridge Institute for Medical Research, The Keith Peters Building, Cambridge Biomedical Campus, Hills Road, CB2 0XY Cambridge, UK; 2 Department of Clinical Neurosciences, University of Cambridge, Cambridge Biomedical Campus, Hills Road, CB2 0QQ Cambridge, UK; 3 John van Geest Centre for Brain Repair, E.D. Adrian Building, Forvie Site, Robinson Way, CB2 0PY Cambridge, UK

**Keywords:** Gene therapy, lentivirus, adeno-associated virus, growth factor, embryonic, pluripotent

## Abstract

Parkinson’s disease (PD) is a neurodegenerative disorder that carries large health and socioeconomic burdens. Current therapies for PD are ultimately inadequate, both in terms of symptom control and in modification of disease progression. Deep brain stimulation and infusion therapies are the current mainstay for treatment of motor complications of advanced disease, but these have very significant drawbacks and offer no element of disease modification. In fact, there are currently no agents that are established to modify the course of the disease in clinical use for PD. Gene and cell therapies for PD are now being trialled in the clinic. These treatments are diverse and may have a range of niches in the management of PD. They hold great promise for improved treatment of symptoms as well as possibly slowing progression of the disease in the right patient group. Here, we review the current state of the art for these therapies and look to future strategies in this fast-moving field.

**Electronic supplementary material:**

The online version of this article (10.1007/s13311-020-00940-4) contains supplementary material, which is available to authorized users.

## Introduction

The long-held promise of gene and cell therapies (GCTs) for Parkinson’s disease (PD) is slowly starting to emerge as a realistic prospect. In the last decade, the first *in vivo* gene therapies (GTs) for non-neurological conditions have met regulatory approval initially in the form of Glybera in 2012 for hereditary lipoprotein lipase deficiency. This was followed not long after by Luxterna in 2018 for the eye disorder Leber’s congenital amaurosis and Zolgensma in 2019 for spinal muscular atrophy [1–3]. The conditions that these therapies are aimed at are however all rare (or “ultra-rare”), and for this reason, such GTs are unlikely to be transformative to substantial healthcare sectors, and their commercial viability has been questioned.

In contrast, PD is a relatively common neurodegenerative disease that, owing to its debilitating long-term effects, carries a large socioeconomic impact. As such, GCTs for PD could be transformative not only for the disease itself but also for the pharmaceutical economy.

Primarily a central nervous system (CNS) disorder, the dominant effects of PD are on movement and cognition, and in early disease motor features correlate tightly with degeneration of dopaminergic neuronal projections from the substantia nigra pars compacta (SNc) to the striatum. Therapeutic responses to pharmacological agents, such as oral dopamine (DA) precursor medications (levodopa), are often satisfactory in the early years after diagnosis, but become steadily less efficacious as the disease progresses. Within 10 years of disease onset, the progression is such that responsiveness to levodopa and other oral dopaminergics is unreliable, and additional DA unresponsive features may begin to dominate the clinical picture. These features can also be aggravated by side effects from the dopaminergic therapies the patient is taking, for example postural hypotension and neuropsychiatric problems.

The problems faced in treating advanced disease, coupled with the predictable progression and anatomically defined nature of the underlying DA deficit, has led to the promotion of GCTs as an attractive prospect for improved symptomatic treatment of PD, and potentially also for disease modification.

## Treatment Options in Advancing Disease

One of the main burdens of living with PD are the fluctuations in motor performance that emerge and then worsen from 5 years after diagnosis, such that after 10 years of the disease, 100% of patients have motor fluctuations, and 56% have levodopa-induced dyskinesias [4]. Loss of striatal dopaminergic innervation is considered a major factor in the development of these clinical features, and DA replacement strategies that more successfully achieve continuous exposure of striatal neurons to DA (such as infusion therapies (IT)), rather than the inconsistent levels provided by oral therapies, have demonstrated a clear ability to smooth out these fluctuations.

The currently widely used ITs (reviewed in [5]) include the following:

- Levodopa–carbidopa intestinal gel (LCIG [6–8]), which is administered by a pump and catheter enterically (intrajejunally), so bypassing gastric emptying.
- Apomorphine, an agonist for DA receptors, which can be administered via subcutaneous infusion, so bypassing the enteric system entirely [9].

Over the last 25 years, however, deep brain stimulation (DBS) has risen rapidly to become the gold standard for treating worsening motor fluctuations in patient who remain levodopa responsive but free of the more severe gait and cognitive issues of advanced PD [10]. By providing round the clock electrical stimulation to the subthalamic nucleus (STN), this technology acts to revert neuronal activity within the movement circuitry from the abnormal pattern that arises with DA depletion, to a pattern more typical of the DA replete (“normal”) state [11]. Using different strategies, therefore, both ITs and DBS achieve a smoother DA response than oral therapies, with consequent beneficial effects on quality of life.

However, each of these options carries its own problems. Apomorphine is frequently poorly tolerated due to side effects, cognitive issues, or local injection site problems. LCIG, often used in patients too elderly or frail to consider DBS, is prone to problems of delivery, for example relating to tube dislodgement or blockage. DBS carries not only immediate surgical risks, but also risks of stimulation-related speech and swallowing difficulties and of device infection. Moreover, each technology has very significant recurring expenses either on a daily basis or in the form of infrequent but expensive hardware maintenance and replacement for DBS.

## A Gap in the Market

Although DBS technologies are still evolving, with promised improvements in targeting, tuning of stimulus effect, and battery life [12], there is nonetheless a “gap in the market” that gene and cell therapies (GCTs) are well placed to fill. An attractive aspect of GCTs is their promise of a one-shot solution, without the down sides and recurring costs of infusion therapies and DBS. Additionally, by being targeted to the predominant site of PD pathology, they may be less prone to the off-target side effects seen with ITs and to a lesser extent with DBS. An ultimately higher goal is the potential for GCTs to slow disease progression, or even to repair aspects of the neurodegenerative changes that occur in PD brain—for example, by using neuronal cell therapies that can release DA in a normal synaptic fashion where it is needed.

From the perspective of GCT technology development, PD has both attractions and hurdles. On the plus side, it is a common condition that is hugely costly to treat by conventional means, and that is also increasingly well understood at a pathological and anatomical level. On the other hand, both ITs and DBS are now well-established and effective treatments, meaning that the bar to improvement is high. As such, the financial risks for companies trying to dethrone these proven technologies are not trivial.

## Vectors for Gene Therapy

Of the various viral vectors that have been investigated over the years, two have emerged as worthy of translation into the clinic for PD. These are adeno-associated viruses (AAVs) and lentiviruses (LVs), and they will each be discussed separately.

### AAV Vectors

Adeno-associated viruses have probably received the lion’s share of attention for use as vectors for GT in PD. They are small (4.7 kB), non-enveloped, single-stranded DNA viruses, of the parvovirus family, and have been considered as potential vectors for gene therapy for over two decades [13–15]. As a family, they are able to infect a wide range of tissues, including both neural and non-dividing cells [16, 17], with long-term transcription of cargo sequences obtained episomally, without integration into host genome [18, 19].

Multiple AAV serotypes exist and have varied tropism for different species and cell populations, meaning different serotypes have been used to target different tissues [20], with the AAV2 serotype in particular showing robust and long-term neuronal expression in both animals and humans ([21–23], reviewed in [14, 24, 25]). The main drawbacks of AAVs are the limited size of transgene they can carry, the ability to upscale their manufacture, and also the theoretical issue of a pre-existing immune response to them in some patients. However, cargo size is only a challenge for certain GT strategies (as will be discussed below), and new methods have now largely resolved previous technical difficulties related to manufacturing large quantities of high quality virus [20, 26].

The immunogenicity of AAVs however remains debated. Although this has not so far emerged as a consistent problem in the immune privileged CNS, it is recognized that AAVs can provoke immune responses that vary with serotype and that may limit the effectiveness of systemic treatments, or prevent repeated dosing [27–29]. Where more serious immune responses have been reported in the brain in animal models, they have been attributed to the non-native transgene in some cases (e.g., intracerebroventricular injection AAV9 with green fluorescent protein [30]). However, it is also possible that the AAV9 serotype is particularly susceptible to detrimental side effects under certain delivery conditions, possibly because of its ability to transfect a wide variety of cells types. Thus, intravenous and intracisternal delivery in animal models has been reported to cause a sensory neuronopathy of variable severity [28, 31, 32]. Interestingly, Perez et al. report that use of species-specific transgene may prevent this outcome, implying that an anti-transgene immune response may contribute in some cases, but autoimmune and toxic causes have also been speculated [28, 32]. The reports have been concerning enough to put a halt to further use of the intrathecal route for delivery of Zolgensma (AAV9) for spinal muscular atrophy—a treatment that received licensing for human use (intravenously) in 2019 [33, 34].

The use of AAV2 in PD has to date not found similar issues, and this serotype has also been employed to carry the *RPE65* gene in Luxterna for eye disease (Leber’s congenital amaurosis) [35–37]. It has also been used experimentally in children, in treatment trials for the rare conditions AADC deficiency [38] and Canavan’s leukodystrophy [39]. Meanwhile, AAV1 has been used in another approved GT, Glybera, for hereditary lipoprotein lipase deficiency HLLD (AAV1) [40].

### Lentiviral Vectors

The second vector type in current use are lentiviruses. These are markedly different to AAVs, being enveloped RNA retroviruses that obtain long-term expression through genomic integration. Retroviruses have long been used in the biological sciences for non-clinical work, owing to their ease of manipulation and also their stable, regulatable expression. The main advantage of LVs over other retroviruses is their ability to infect postmitotic cells, including neurons, which facilitates their use as vectors for neurons *in vitro*. This has made them attractive candidates for use in CNS diseases.

The best known lentivirus is the human immunodeficiency virus (HIV), but the first to be taken forward towards human GT use was a non-human LV, the equine infectious anemia virus (EIAV). This was engineered to include minimal viral sequences, with proteins required for making up the viral particle (Gag) and replicatory enzymes (Pol) being supplied “in trans” in the packaging process, so not incorporated in the genome of the resultant particles [41]. These engineered viruses are thus replication defective and contain less than 10% (or < 1kB) of the original viral genome; the only protein expressed by the integrated DNA within the target cell is the cargo protein of choice—from the transgene cassette. The tissue tropism of LVs tends to be quite broad, and they have proven to be less flexible than AAVs, but the engineered EIAV in current use is fully neurotropic, being pseudo-typed with the envelope protein of vesicular stomatitis virus (VSV-G), which facilitates a broad tissue (and species) tropism. Other pseudo-typing has been used with different tissue tropisms [42, 43], but for fuller tissue specificity, LVs have the cargo capacity to include tissue-specific enhancers [44], something not possible in AAVs.

A theoretical concern with LVs is that the genomic integration that is integral to their function and also provides a potential for oncogenicity. Such oncogenicity has been seen with some GTs, but only for retrovirus-mediated gene delivery to bone marrow populations for immunodeficiency disorders [45–48]. The key difference here from the EIAV technology is that the viruses employed were not LVs, but γ-retroviruses, which have a distinct mode of integration into the host DNA that makes them more prone to inducing oncogenicity [49]. In addition, the transduced cells were actively proliferating, rather than postmitotic, and so are probably given a selective growth advantage, promoting the development of additional mutations and oncogenicity. Such considerations make it unlikely that LV transduction of CNS neurons will provoke oncogenesis, although some uncertainty around this issue must remain.

## Gene Therapy Strategies

**Fig. 1:**
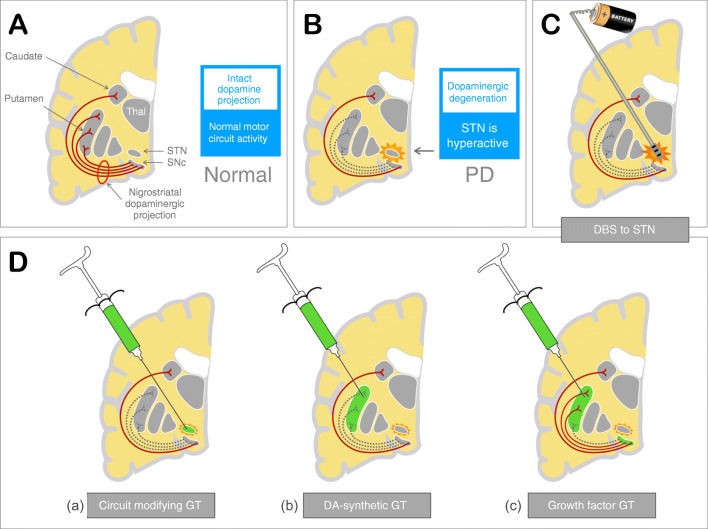
Rationale for the different gene therapy strategies currently used in PD, compared with deep brain stimulation. (A) Normal motor circuit anatomy and activity. (B) In PD, there is degeneration of the nigrostriatal (NS) projection from the substantia nigra pars compacta (SNc) to the putamen. This results in abnormalities of motor circuit activity, including hyperactivity of the STN nucleus. (C) DBS stimulation of the STN inhibits STN hyperactivation and so restores normal circuit activity. (D) Normal motor circuit function can be restored through GT: (a) *Circuit modifying GT* uses gene transfer of the enzyme GAD to the STN, so directly inhibiting STN activity. (b) *DA synthetic gene GT* replenishes DA supply within the putamen, so normalizing circuit activity and function. (c) *Growth factor GT* exposes axonal terminals of NS projection neurons within the putamen to GFs, so facilitating transport of such GFs to the SNc. This may prevent further degeneration of the NS projection and boost function of residual axonal arbors.

The target of the current GTs for PD is the motor circuitry that is deprived of dopaminergic innervation as a result of the degeneration of the nigrostriatal (NS) projection. There are however several potential approaches by which GT can be used to manipulate this pathological circuitry, so improving patient symptoms. The three main strategies are described below (see also Fig. 1):

1. *Gene therapy to modify motor circuitry*: circuit modification by GT uses gene delivery to a critical node within the motor circuitry of the basal ganglia to provide a biological effect similar to DBS.
2. *Gene therapy to synthesize DA*: DA-synthetic GT aims to restore the DA synthesis capacity in the tissue where it is needed, in this case the putamen—the major target of the degenerating NS projection.
3. *Gene therapy to modify disease*: growth factor (GF) GT uses GFs to boost survival and function of residual NS neurons, so slowing disease progression in this pathway and supporting residual DA production.

Each of the approaches has potential benefits and drawbacks, and all have now been trialled in patients with PD. For each, AAV and LV vectors might be considered as closely equivalent options, given that both have demonstrated long-term expression in humans over periods of years—up to 4 years for LVs in ocular gene transfer of endostatin/angiostatin for macular degeneration [50], and over 10 years according to postmortem data on AAVs in PD [42]. AAVs are though the more established technology, having been used in the majority of trials to date. LVs have so far been used exclusively for one of two DA-synthetic approaches, but if regulatable expression becomes useful or necessary in due course, then they may well find roles in other areas [51].

## Gene Therapy to Modify Circuitry

The DA depletion that characterizes PD results in profound changes in the motor circuit of the basal ganglia and related structures. These changes derive from DA’s role as a modulator of neuronal activity (neuromodulator), which affects different pathway activities in distinct ways. For example, the direct and indirect pathways are respectively activated and inhibited by DA, resulting in loss of the normal balance of activity between these two pathways as DA levels decline. Allied to this, the subthalamic nucleus (STN), a key node in the circuit, shows heightened synchronized activity in the absence of DA, with a pattern that is dominated by low frequency neuronal spiking [52, 53]. This synchronized hyperactivity, so-called β band activity, is felt to be specifically detrimental to motor performance, being tightly correlated with some clinical features of PD, including slowness (bradykinesia), stiffness (rigidity), and tremor. The ability of DBS to interfere with β band activity may be integral to how it achieves its beneficial effects [11] (Fig. 1C).

The use of AAV2 to deliver glutamic acid decarboxylase (GAD) to the STN is a logical extension of these insights. The STN has a physiological output that is glutamatergic and therefore excitatory to its downstream target nuclei. By transfecting STN neurons with a gene for GAD, which converts the excitatory neurotransmitter glutamate to the inhibitory transmitter gamma-aminobutyric acid (GABA), the output of the STN can in principle be converted from excitation to inhibition. This is predicted both to dampen activation of STN target nuclei—the globus pallidus interna (GPi) and substantia nigra pars reticulata (SNr)—and also, through effects on reciprocal interactions within the STN, to suppress synchronized activity of the nucleus itself. In essence, the strategy promises to achieve through GT a similar benefit to DBS, but without the need for long-term implantation of batteries and wires. The aim of this form of therapy is illustrated in Fig. 1D(a), and the relevant clinical trials are summarized in Table 1.

**Table 1:** Summary of circuit modifying GT trials to date

NCT number, sponsor, and site	Virus and dosing	Study details and outcomes
NCT00195143 Sponsor: Neurologix Site: Cornell, NY Duration: 6 months Status: completed Refs: [54]	AAV2-GAD65/GAD67 STN, unilateral Low dose: 4.5 × 10^9^ vg/STN Medium dose: 1.4 × 10^10^ vg/STN High dose: 4.5 × 10^10^ vg/STN Infusion: 45 μl/STN iMRI—no	Category: phase 1, safety and dosing Patients: 12 subjects Disease stage: advanced PD; avg. duration 9 years Primary: safety. Well tolerated, no AEs Secondary: improvement in UPDRS, mainly on treated side; reduction in thalamic metabolism on PET on treated side
NCT00643890 Sponsor: Neurologix Site: Multicenter Status: Terminated (financial) Duration: 6 months Refs: [55–57] Long-term f/u: NCT01301573	AAV2-GAD65/GAD67 STN, bilateral Dose: 4.5 × 10^10^ vg/STN Infusion: 45 μl/STN iMRI—no	Category: phase 2, randomized, sham surgery Patients: 45 patients—split 22/23 (active/sham) Disease stage: advanced PD; avg. duration 10.6/12.0 years Primary: UPDRS scores Secondary: multiple including ^18^F-FDG

This approach was pioneered by a collaboration between investigators at Weill College of Medicine at Cornell and a biotech company Neurologix, and this group were in fact the first to use GT to treat adult neurological disease. Early preclinical studies in rodents [16, 58], and later in primates [59], were encouraging, showing correction of clinical deficits and even a degree of neuroprotection. This data facilitated subsequent clinical studies, and a phase 1 safety study of unilateral STN treatment using viruses carrying a mix of genes for two GAD isoforms (GAD65 and GAD67) was published in 2007 (NCT00195143) [54]. Three dose cohorts were used in 12 patients with PD with an average disease duration of around 9 years. AAV2-GAD was infused into the STN unilaterally in an open-label study design. The results showed the procedure to be well tolerated, with no adverse events (AEs) related to the intervention. There were also statistically significant changes in UPDRS scores and FDG-PET signal on the treated side.

With this encouraging data, Neurologix and the study team went forward to a phase 2 double-blinded study of bilateral STN injections, recruiting 45 patients split (23/22) between sham and a treatment arms (NCT00643890) [55]. Again the procedure was well tolerated, with no AEs related to treatment. However, this time, in the blinded format, efficacy results were disappointing. Improvement in the defined OFF UPDRS part 3 motor scores at 6 months were significant, but only modest at 23% compared with 12% in the sham arm. For comparison, the first randomized controlled trial using DBS showed a 41% improvement in OFF UPDRS part 3 score at 6 months [10]. For this reason, the Neurologix trial was not considered a resounding success, and Neurologix later filed for bankruptcy in 2012.

This strategic failure underlines the high bar to success that exists in the form of STN-DBS, the current gold standard competitor in this therapeutic space. However, this may not be the end of the road for STN-GAD therapy, as a subsequent analysis has emphasized a real and potentially useful effect, with clinical improvements persistent at 12 months and matched by measurable and correlatable changes in network activity as seen with FDG-PET imaging [56, 57, 60]. These later results have yet to salvage the approach, but the technology has now been obtained by another biotech company, MeiraGTx, and is still currently considered to be one of their pipeline products for the treatment of PD. Its potential advantage over other GT strategies lies in its relative ease of administration: the STN is a small and well demarcated target that functional neurosurgeons target frequently for DBS; crucially, it could be infused with the relevant vector more quickly and completely than the larger putamen (see below). However, for the same reason, off-target effects may be more difficult to control.

## Gene Therapy to Synthesize Dopamine

Within the basal ganglia, DA is delivered to the striatum by axonal projections from the substantia nigra (SNc), which thus regulate striatal function and motor and cognitive performance. In the striatum, the main population of striatal output neurons—medium spiny neurons (MSNs)—are exposed to DA in both “tonic” and “phasic” modes, with phasic release being tightly correlated with circuit activity and behavior, and important for habit learning [66]. Phasic DA responses cannot be reconstructed by gene delivery to the striatum, but tonic DA exposure seems sufficient to ameliorate many of the features of PD in both patients and animal models. Replenishment of DA supply through GT is therefore an obvious approach. To date, two vector/cargo combinations have been employed to achieve resupply of DA to the DA-denervated putamen. The aim of this therapy is illustrated in Fig. 1D(b), and the relevant clinical trials are summarized in Table 2.

**Table 2:** Summary of dopamine synthetic GT trials to date

NCT number, sponsor, and site	Virus and dosing	Study details and outcomes
NCT00229736 Sponsor: Genzyme Site: University of California Duration: 6 months Status: completed Refs: [61]	AAV2-AADC Putamen, bilateral Low dose: 9 × 10^10^ vg/patient High dose: 3 × 10^11^ vg/patient Infusion: 2 deposits of 50 μl per putamen iMRI—no	Category: phase 1, safety and tolerability Patients: total 10 subjects, 5 per dose Disease stage: advanced PD; avg. duration 13/9.4 years Primary: safety. Well tolerated. One patient with symptomatic hemorrhage, almost complete recovery. Two with asymptomatic hemorrhages Secondary: improvements in UPDRS, Hauser diaries, and FMT-PET
NCT: no number Sponsor: Genzyme Site: Jichi University Duration: 6 months Status: completed Refs: [62]	AAV2-AADC Putamen, bilateral Dose: 3 × 10^11^ vg/patient Infusion: 2 deposits of 50 μl/putamen iMRI—no	Category: phase 1, safety and tolerability: Patients: 6 patients Disease stage: advanced PD; avg. duration 10 years Primary: safety. Well tolerated. One patient with small hemorrhage and transient symptoms. Secondary: improvements in UPDRS and ^18^F-m-tyrosine PET
NCT01973543 Sponsor: Voyager Therapeutics Site: Univ. of California and Univ. of Pittsburgh Duration: 12 months Status: completed Refs: [63]	AAV2-AADC (VY-AADC01) Putamen, bilateral Low dose: 7.5 × 10^11^ vg/patient Medium dose: 1.5 × 10^12^ vg/patient High dose: 4.7 × 10^12^ vg/patient Infusion: iMRI/SmartFlow, 2 or 3 tracts, 450–900 μl/putamen iMRI—yes	Category: phase 1, safety /dose finding Patients: 15 subjects, 5 per dose Disease stage: advanced PD Primary: safety. Well tolerated. One patient with three SAEs, due to immobility during procedure—DVT, PE, AF. Secondary: improvements in all—putaminal coverage (iMRI); ^18^F-DOPA PET at 6 months; OFF UPDRS3 at 12 months; PDQ-39; levodopa dose
NCT03562494 (RESTORE-1) Sponsor: Voyager Therapeutics Site: multicenter Study duration: Status: closing 2020 Refs: not yet published	AAV2-AADC (VY-AADC02) Putamen, bilateral Up to 2.5 × 10^12^ vg/patient Infusion: 2 or 3 tracts, up to 900 μl/putamen iMRI—yes	Category: phase 2, blinded RCT, sham surgery control Patients: 42 subjects Disease stage: advanced PD Primary: safety (multiple, including psychiatric); striatal coverage; diary; PET (^18^F-DOPA PET) Secondary: pending
NCT00627588 Sponsor: Oxford Biomedica Site: Paris (France) and Cambridge (UK) Duration: 12 months Status: completed Refs: [64, 65]	EIAV-triple enzyme (ProSavin®) Putamen, bilateral Dose: three dose levels, 1·9 × 10^7^, 4.0 × 10^7^ and 1.0 × 10^8^ transducing units (TU)/patient iMRI—no	Category: phase 1, safety and dose finding Patients: 15 subjects, 3 dose levels. Disease stage: advanced PD; avg. duration 13.9 years [8–27] Primary: safety. Well tolerated; no SAEs due to treatment Secondary: improvements in UPDRS 3 and PDQ-39; modest improvements in raclopride-PET, and LED
NCT03720418 (SUNRISE-PD) Sponsor: Axovant Site: Paris (France), Cambridge (UK), and National Hosp. (London) Duration: ongoing Status: active Refs: not yet published	EIAV-triple enzyme (OXB-102) Putamen, bilateral Dose: three dose levels, 4.2 × 10^6^, 1.4 × 10^7^, and 4.2 × 10^7^ TU/patient Infusion: 3 deposits of 100 μl/putamen iMRI—no	Category: phase 1/2—safety and dose finding, moving to sham surgery Disease stage: advanced PD Patients: 3–4 patients per dose, 3 dose levels Primary: safety Secondary: UPDRS, Hauser diaries, PET, dyskinesia rating scale; Hauser diaries; PDQ-39, SF-36, and clinical global impression

### AAV2-AADC

The first of the two DA synthetic strategies aims to restore sensitivity to exogenous levodopa treatment by augmenting striatal levels of the enzyme aromatic L-amino acid decarboxylase (AADC). AADC is the final step in conversion of levodopa to DA, and its striatal expression within the dopaminergic nerve terminals is lost with disease progression [67, 68]. Thus, although not a dose-limiting step physiologically, its levels become dose-limiting with disease progression, and AAV-mediated transfection of AADC into striatal MSNs should theoretically correct this deficit. Restoring AADC activity then allows striatal DA to be synthesized in response to exogenous levodopa, which must be supplied orally [68, 69]. This strategy has the benefit of retaining some control over the amount of DA synthesized, via alterations in oral levodopa dose. A single AADC gene is well within the cargo size of the AAV vector, and preclinical studies in rodents and parkinsonian non-human primates (NHPs) have paved the way for using AAV to deliver its AADC cargo to patients in the clinic [70, 71].

The first studies of AADC gene therapy in patients with PD were performed in California under the sponsorship of the biotech company Genzyme (NCT00229736) [61, 72]). Using an AAV2 vector, the group completed a twin-dose safety study in 10 patients with moderately severe disease (average disease duration of 11.2 years). Two cohorts of 5 patients were split into low and high doses that equated to 9 × 10^10^ viral genomes (vg) and 3 × 10^11^ vg per patient, administered via two injection sites at a volume of 50 μl per putamen. From a safety perspective, the treatment was well tolerated over the 6 months of the study, although three of the patients had small intracerebral hemorrhages along the trajectory of the catheter at the time of surgery (two of which were asymptomatic). Secondary outcomes included clinical assessments in the defined OFF medication state, and these showed significant (up to 30%) improvements in both cohorts, whereas diary scores improved in all patients, and levodopa daily dose was reduced in three of the patients in the low dose cohort, and all five in the higher dose cohort. There were also significant increases in ^18^fluoro-L-m-tyrosine (FMT)-PET signal in both cohorts, rising to 75% of baseline in the high dose subjects.

These initial results were encouraging and were also corroborated independently by a group in Jichi University, using an identical vector and methods [62]. Six subjects received the same dose as the high dose patients from California, and again the procedure was well tolerated, with no sustained AEs, although one venous hemorrhage at the site of a cannula insertion produced minor transient motor deficits. Again, there was an apparent benefit as assessed by a number of secondary outcomes at 6 months, with a 46% improvement in defined OFF UPDRS part 3 motor scores and a 56% improvement in FMT-PET signal.

Due to concerns about tissue (putaminal) coverage, partly sparked by data from the contemporaneous neurturin (NRTN) trials (see below), these phase 1 trials were followed by a refined phase 1 study, again with a primary focus on vector delivery, dose finding, and safety (NCT01973543) [63]. Now under sponsorship of the biotech company Voyager Therapeutics, a novel delivery technique was introduced which aimed to optimize tissue coverage. This employed a new stepped tip cannula (SmartFlow, MRI Interventions, Inc.) and a novel surgical targeting system (SmartFrame and Clearpoint neuro-navigational system - MRI Interventions, Inc., Irvine, CA), working in conjunction with the Brainlab software (iPlan Flow). Intraoperative MRI (iMRI) and the contrast agent gadolinium (Gd) facilitated visualization of putaminal coverage in the operating theatre. This in turn allowed the use of higher volumes of infusate and also flexibility in the number of cannulae employed (two or three per side), so optimizing coverage. Using this approach, previous volumes of 100 μl per putamen were shown to be inadequate, and were therefore increased up to as much as 900 μl per putamen, with viral doses being increased incrementally in 3 cohorts, up to 4.7 × 10^12^ vg per subject (50 times the low dose of the original study).

Results again showed the procedure to be well tolerated [63]. One subject suffered three serious AEs (SAEs) consisting of a deep vein thrombosis and pulmonary embolus and subsequent atrial fibrillation, all of which resolved and were felt to be linked to the immobility needed for the infusion rather than the agent itself. In the higher dose cohort, putaminal coverage was up to 42% by iMRI, with a 79% increase in baseline ^18^F-DOPA PET signal. Secondary endpoints included UPDRS part 3 motor ON scores, UPDRS part 3 defined motor OFF scores, Hauser diary entries, levodopa equivalent dose (LED), and PDQ-39. All of these measures showed improvements across the three dosing cohorts, with dose dependency present for all except diary scores. There were particularly impressive reductions in daily levodopa equivalent dose (LED), which in the high dose cohort was similar (at − 41%) to that obtained by DBS. Mild transient increases in dyskinesia were noted at 1 month, but the therapy was broadly well tolerated, in particular with no behavioral AEs.

Although encouraging both in terms of the degree of improvement and persistence over the time of the study (up to 36 months), these results do not exclude a significant placebo effect. Thus, the current status of AAV2-AADC technology is that Voyager Therapeutics is undertaking a multicenter phase 2 study (RESTORE-1), using a slightly modified virus at a single dose level, in a sham surgery-controlled trial. A heavy emphasis in this study is being placed on safety, including psychiatric parameters, but primary outcomes may now also include striatal coverage by MRI, F-DOPA PET imaging, and diary scores. All being well, the company hopes to push forward towards a phase 3 study in the near future (RESTORE-2) [73].

### EIAV-Triple Enzyme

The direct competitor to the AADC therapy is the more comprehensive “triple enzyme” approach [64, 65]. Again, the anatomical target is the striatum, but in this format, the viral cargo encodes all three enzymes required to synthesize DA from the precursor amino acid tyrosine. These include tyrosine hydroxylase (TH), GTP cyclohydrolase 1 (GCH1, an enzyme required for synthesis of tetrahydrobiopterin, a cofactor for TH), and also AADC. The inclusion of all three enzymes is aimed at facilitating a more definitive reduction in medication doses than the AADC approach. Additionally, with no reliance on an adequate biodistribution of exogenous levodopa, the strategy could facilitate a more efficient elimination of motor fluctuations. Coding for three enzymes is however beyond the carrying capacity of AAV-based vectors, and so this strategy has utilized a LV vector initially developed by the biotech company Oxford Biomedica [74, 75].

Initial preclinical work demonstrated the practicality and safety of using an EIAV LV for CNS gene therapy in both rodent and NHP models of disease [41, 75–79]. The first in human use of a LV gene therapy to the CNS was then undertaken, initially in Paris and then Cambridge, UK, by Oxford Biomedica using their ProSavin® vector [64]. Patients had advanced disease (average duration 13.9 years), and dosing was between 1.9 × 10^7^ and 1.0 × 10^8^ transducing units (TU) per patient. Surgical infusion was performed via five tracts per putamen initially, but this was simplified to three tracts in the final surgical format. Safety outcomes in the initial 15 patients showed no AEs related to the agent, whereas secondary measures of efficacy showed improvements relative to baseline at 6 and 12 months in the UPDRS part 3 motor score (32% and 29%) and also in PDQ-39 (− 4.9 points, − 14.7%) at 6 months. Dose reductions in LED were however modest, matching those changes seen on Raclopride PET binding (− 5.2%, − 9.6%, and − 10.1% in low medium, and high dose cohorts).

This good safety record and hints at efficacy serve to reinforce the AAV data and emphasizes the broad potential for surgically delivered GTs in PD. The unblinded data from the LV/ProSavin approach [64] is perhaps slightly less convincing than for the Voyager AAV-AADC study published more recently [63], but like the original Genzyme study, the ProSavin study was a first attempt and has set the scene for a further dose-finding and safety trial with higher doses of vector. This new trial, Sunrise-PD, is currently underway and may produce more convincing effects.

Sunrise-PD addresses concerns of suboptimal production of the three enzymes through a vector that has been re-engineered to optimize DA production [80]. Specifically, the TH gene, already truncated to avoid auto-inhibition, is now expressed as a fusion protein with GCH1, so optimizing expression and allowing for a single internal translational initiation site (IRES) upstream of the second cassette that contains the AADC gene. Published data suggests that DA production in transfected cells will now be 5–10 times higher than with the previous version [80]. As with the original trial, delivery of the vector (OXB-102) is by infusion, but will now rely on 3 injections of 100 μl per putamen (total 300 μl per side). Sunrise-PD commenced at the end of 2018 and is now sponsored by Axovant Gene Therapies. Once dose optimization is complete, a move towards a planned sham surgery phase is likely to happen in 2021.

### Realistic Competitors for DBS

Although AADC and triple enzyme gene therapies do not represent a cure for PD, or even a slowing of disease progression, they do represent a potentially viable alternative to DBS, the current gold standard for treating motor fluctuations. As described above, the target patients in trials to date have been those with moderately advanced PD and motor fluctuations, which is exactly the group in which DBS has found its major niche.

Whether there is room for both of these two new gene therapies in this space, alongside DBS and infusion therapies, is not clear at present. How things work out is likely to depend on just how successful each modality is in improving fluctuating motor features in this problematic patient group. The triple enzyme approach, which is not reliant on a supply of oral levodopa, has the potential to achieve a greater reduction in oral medication than AAV2-AADC and a better smoothing out of motor fluctuations. In practice, how much this distinction really matters is yet to be understood, and the AADC approach may yield a lower risk of uncontrolled motor or neuropsychiatric side effects, because the DA stimulus can be regulated not only by viral dose and tissue distribution, but also by oral levodopa dose after surgery. Moreover, in the higher dose cohorts in the Voyager/AADC trial, subjects not only benefited from reduced fluctuations but also managed to reduce their oral levodopa intake by very significant amount and still achieve improved OFF medication scores on formal motor testing [63]. This suggests that increasing AADC enzyme levels in the striatum may improve some symptoms even in the absence of a recent oral levodopa dose (perhaps by improving utilization of residual endogenous levodopa).

### Role of iMRI Imaging

At present, the other distinction between the two approaches is turning out to be the reliance of the AAV2-AADC approach on iMRI to monitor the vector distribution obtained by their convection enhanced delivery (CED) technology. The Voyager team has focused heavily on this aspect, using Gd-iMRI monitoring as a means of optimizing vector delivery at the time of surgery.

The technique of CED was originally described in the 1990s [81, 82] and relies on generation of a pressure gradient at the needle tip that enhances tissue spread of vector by bulk convective flow rather than by diffusion. It has been pioneered by the Bankiewicz team and is now in use in both AAV2-AADC and AAV2-GDNF trials (see below). Using a 1:1 mix of gadolinium contrast agent (Gd) and viral sample, the distribution of viral infusate can be accurately monitored in real time using iMRI. NHP studies have confirmed a tight match between MRI visualization and pathological distribution of vector expression [83], and needle design has incorporated a shoulder into the device, a short distance back from the exit point of the needle, to reduce backflow and optimize the pressure gradient [84].

This strategy promises optimization of effect in individual patients, but more importantly it may also facilitate refinement of surgical technique over time. Thus its benefits are already apparent in enabling safe administration of higher volumes of infusate than are planned for the Axovant/LV study. Indeed, such high volumes may well partly underlie the promising outcome data in the latest unblinded Voyager study [63]. However, visualization of tissue coverage also promises a greater understanding (and avoidance) of off-target effects, which could turn out to be extremely important, particularly as continuous dopaminergic stimulation of ventral striatum carries the risk of unwanted neuropsychiatric side effects.

Although the LV strategy has not yet used iMRI to monitor tissue distribution, it is unclear whether or not this is a true drawback. There are differences in how the two vectors move through brain tissue that may make iMRI less useful for LVs (AAV2 diffuses poorly, so must be convected [83]), and lack of a need for iMRI technology could boost uptake of the EIAV-triple therapy technology, in the context that many DBS centers outside North America do not currently employ iMRI in their DBS practice. This might give the LV vector a competitive advantage, at least in the short term. One current limitation to comparing these two approaches is the lack of any postmortem data on EIAV vector distribution in humans, and so the field relies heavily on NHP data. Ultimately, whether the LV strategy adopts some form of iMRI monitoring in due course remains to be seen.

## Gene Therapy to Modify Disease

### Growth Factors in PD

The extensive demonstrations of safety and efficacy of GT in late PD, by the twin strategies of DA replacement and circuit modification, is very important for the future of the technology in both PD and other CNS diseases. However, in reality, these approaches only offer a better means of managing motor features in a disease that is already advanced and irreversible. Given the wealth of historical data examining DA neuronal survival, a more ambitious strategy would look to use GT strategies to slow or halt the DA neuronal loss that underlies disease progression.

Growth factors (GFs) have a long history of exploration in PD (reviewed in [89]), having been thoroughly examined in both cell culture and PD animal models over several decades. Particularly, promising beneficial effects have been noted for two related GFs: glial cell line derived neurotrophic factor (GDNF) and neurturin (NRTN) [90, 91]. Both of these are able to enhance survival and neurite outgrowth of DA neurons *in vitro* and *in vivo* [92–95], and both can rescue DA neurons in preclinical models of PD [78, 96–98]. Such work has raised hopes for using these GFs as therapeutic agents in PD, both to slow DA neuronal loss and to enhance function in the surviving DA neurons and their axon terminals [99, 100]. The aim of this form of therapy is illustrated in Fig. 1D(c), and the relevant clinical trials are summarized in Table 3.

**Table 3:** Summary of GF GT trials to date

NCT number, sponsor and site	Virus and dosing	Study details and outcomes
NCT00252850 Sponsor: Ceregene Site: Univ. California, San Francisco, and Rush, Chicago Duration: 12 months Status: completed Refs: [85]	AAV2-NRTN (CERE-120) Putamen, bilateral Low dose: 1·3 × 10^11^ vg/patient High dose: 5.4 × 10^11^ vg/patient Infusion: 8 × 5μl deposits/side, split between 4 needle tracks/side iMRI—no	Category: phase 1, safety and dosing Patients: 12 subjects Disease stage: advanced PD; avg. 11 years Primary: safe and well tolerated Secondary: no significant benefits in OFF UPDRS3 at 12 months and ^18^F-L-DOPA PET at 6 and 12 months
NCT00400634 Sponsor: Ceregene Site: multicenter Duration: 12 months Status: completed Refs: (86)	AAV2-NRTN (CERE-120) Putamen, bilateral Dose: 5.4 × 10^11^ vg/patient Infusion: 8 × 5μl deposits/side (40 μl) 4 needle tracks/side, 2 deposits/track iMRI—no	Category: phase 2, randomized, double-blind, sham-surgery–controlled Patients: 58 subjects–38/20 in surgery/sham arms Disease stage: advanced PD; avg. 9.5/10.0 years, in active/sham arms Primary outcome: nonsignificant improvements over sham surgery in OFF UPDRS3 Secondary outcome: nonsignificant trends to improvement UPDRS; timed motor tests; dyskinesia rating scale; diaries; PDQ-39, SF-36, and clinical global impression
NCT00985517 Sponsor: Ceregene Site: multicenter Duration: 15–24 months Status: completed Refs: (87)	AAV2-neurturin (CERE-120) Putamen and SN, bilateral Dose: 2.4 × 10^11^ vg/SNc; 1.0 × 10^12^ vg/putamen Infusion: two deposits of 15 μl/SNc; one deposit of 50 μl in each of 3 putaminal tracts iMRI—no	Trial category: phase 2, randomized, double-blind, sham-surgery–controlled Patients: 48 patients, 23/25 treated/sham arms Disease stage: advanced PD; avg. duration 7.8/8.6 years in treatment/sham arms Primary: no significant change in OFF UPDRS part 3 at 15 months Secondary: nonsignificant changes in other UPDRS, diaries, PDQ-39
NCT01621581 Sponsor: NIHCC Site: Bethesda Status: active, NR Duration: 18 months Refs: (88)	AAV2-GDNF Putamen, bilateral Low dose: 4.5 × 10^10^ vg per putamen Medium dose: 1.5 × 10^11^ vg/putamen High dose: 4.5 × 10^11^ vg/putamen 450 μl/putamen iMRI—yes	Trial category: phase 1, safety and dose finding, 4 dose levels Patients: 25 subjects; three dose levels Disease stage: > 5 years disease Primary: safety and tolerability. Well tolerated. Six SAEs, none attributable to study drug; all resolved Secondary: Nonsignificant improvements in ^18^F-F-DOPA PET at 18 months. No significant change in UPDRS, LED at 6/18 months; PET scan at 18 months
NCT04167540 Sponsor: Brain Neurotherapy Bio, Inc. Site: University of California NYR Ohio, Duration: TBA Status: recruiting REFS: clinicaltrials.gov	AAV2-GDNF Putamen, bilateral iMRI—yes	Trial category: phase 1, safety Patients: 12 subjects, 2 cohorts Disease stage: earlier (< 5 years) and later (> 5 years, moderate–severe disease) Primary: safety tolerability over 5 years Secondary: UPDRS, nonmotor scores, and DAT scan changes over 18 months

### Protein Infusion—GDNF

The first in human use of GFs to treat PD was not through GT, but via surgical infusion of the recombinant GF protein (rGDNF) itself, which is relevant to briefly review. A sequence of trials were made, starting with the unsuccessful use of intraventricular rGDNF delivery [101], before moving on to procedures that targeted the putamen directly. These initial open-label intraparenchymal infusion studies showed some promising outcomes [102–104]. However, a randomized study, using infusion pump driven delivery of rGDNF to the putamen, failed to meet its primary trial outcomes at 6–8 months [105].

Based on methodological concerns, additional evidence of benefit from postmortem studies, and anecdotal longer term follow-up data on some patients from the initial open-label trials [106, 107], a revised double blind placebo-controlled trial was undertaken in Bristol, UK, and the results from this have been published recently [108, 109]. This trial abandoned pumps, using instead intermittent (monthly) infusions of rGDNF, for up to 80 months. A stronger emphasis was also put on tissue coverage, and a MRI-monitored CED infusion approach was employed. Although the trial did not meet its primary end point, improvements in ^18^F-DOPA PET were documented, and 95% of the patients receiving rGDNF for the full 80 weeks gained a meaningful clinical improvement in one or more of the core outcome measures. Whether this constitutes a useful outcome is unclear, but the surgical delivery system employed in this study was complex and not without complications, as has also been reported in the new ongoing trial of recombinant cerebral dopamine neurotrophic factor (rCDNF) protein being undertaken in Scandinavia [110]. As such, these recombinant GF protein therapies are probably best considered as preparing the ground both for the more tractable genetic approach of GF GTs.

### AAV2-NRTN—Ceregene

Despite earlier work using LV-mediated GDNF delivery in animal models, the first GF to come to the clinic in the format of a GT was actually NRTN rather than GDNF, and used an AAV2 vector, rather than LVs. AAV2-NRTN was manufactured by the Californian biotech company Ceregene, with their product CERE-120 providing neuronal expression of NRTN as a pure protein without viral coding sequences [111]. To enhance secretion, the preprodomain of human nerve growth factor was used in place of the NRTN sequence, and preclinical testing showed CERE-120 to be well tolerated, with persistent gene expression for periods of over 12 months and minimal biodistribution beyond the target area [111–113] (reviewed in [114]). Therapeutically, it showed promising effects on the survival of SNc DA neurons in rodent and NHP PD models.

Initial clinical studies followed, and a phase 1 study of 12 patients, with an average disease duration of 11 years, found CERE-120 to be well tolerated (NCT00252850) [85]. This study was not powered to determine whether the agent had either a symptomatic benefit or a later disease-modifying effect, but even so ^18^F-levodopa PET findings were a little disappointing, with no clear change in striatal signal.

The real stumbling block was the subsequent blinded study (NCT00400634), which recruited 38 patients to the active arm and 20 patients to sham surgery [86]. Notably, the average disease duration in the recruited participants was 9.5 and 10 years in the surgical and sham arms respectively, and by this measure, subjects were slightly more advanced than those in the GDNF protein infusion studies [105, 109]. A viral dose of 5.4 × 10^11^ vg per brain was comparable to other putaminally targeted AAV2 procedures, but this dose was delivered without MRI guidance, and as eight separate deposits, each of 5 μl, split between four needle tracts per hemisphere (total 80 μl). The trial failed to meet its primary end point of a change in the UPDRS part 3 motor score in the defined OFF medication state at 12 months with the intervention.

On the positive side though, the procedure was again well tolerated—SAEs occurred in both arms, but none were attributable to the novel therapy. However, a failure to meet the primary endpoint meant that the therapy as delivered could not be competitive with the established technology of DBS. Things could have stopped there, but as with the Bristol GDNF protein study [109], there were clues in the original data that a subset of patients might be getting significant benefit, with patients assessed at 18 months demonstrating a significant benefit over placebo. This was not the primary endpoint, and the benefit was only seen in a small subset of the total patients, but this and some additional trends to improvement with surgery in some of the secondary endpoints suggested that modifications to the delivery might yet improve outcomes over a longer time course (see below).

### AAV2-NRTN—Postmortem Data

An important element in this decision to continue trialling this agent was postmortem data on four patients, who died of unrelated causes subsequent to receiving the gene therapy, which lent more insight into the disappointing outcome. Two of these individuals died within a few months of treatment and two of them about 4 years later, so giving a time course of transgene effects. There were three main findings from these pathological studies [115, 116], the first being the technical issue of poor tissue coverage by the GT within the putamen. Thus, although putaminal NRTN expression evoked by the treatment was good and persistent, the volume coverage per infusion was less than 20% of total putaminal volume, which was probably insufficient to provide good clinical benefit.

The second observation was that dopaminergic denervation of the striatum was surprisingly advanced even at 5 years into the disease. Probably at least partly as a result of this, the enhanced tyrosine hydroxylase (TH) expression evoked by the NRTN was rather modest and only covered ~ 2% of striatal area in the short-term cases and 13.4% in the longer term (4 years) cases. This suggested a *resistance* to trophic effect in these patients, albeit one that was partly overcome with time.

Thirdly, the striatal NRTN expression was not matched by similar NRTN expression in the SNc as would be expected by the retrograde transport of GF observed in preclinical models. This was improved in the two longer term patients but was still poor even compared with aged animal models and was matched by deficient transport to other putaminal targets such as GPi. This suggests a retrograde axonal transport deficit in patients with PD, which may be specific for certain projections, as the transport of NRTN from putamen to cortex (via axon terminals within the putamen) seemed to be largely intact.

Together, these findings imply that the degenerating human NS system offers a significantly different environment for disease-modifying strategies than is apparent in either preclinical rodent or NHP PD models, and that both better tissue distribution of AAV2-NRTN and more protracted time points might be needed. More fundamentally, it is difficult to envisage a GF working on NS projections that no longer exist, and data from other groups also suggests that the α-synuclein pathology of PD may itself impair both axonal transport and GDNF/NRTN receptor (RET) expression and signalling in the surviving NS projections [117–119]. This has been debated [120], as has the extent to which there may be unwanted detrimental effects of these GFs [121].

### AAV2-NRTN—a Revised Attempt

The logical outcome from this pathological data was that NRTN delivery to relevant tissues still needed to be optimized. This would require several improvements on existing methodology, including larger volumes of infusate, delivery of vector to both putamen and SNc, and inclusion of earlier stage patients. Moreover, assessment would need to be done over longer time points than the 12 months of the original studies. Revised studies were therefore embarked up on which partly explored these issues, specifically by reinforcing putaminal expression of NRTN with co-expression of the same vector in SNc. This line of investigation required further preclinical studies, which ultimately concluded in a revised blinded sham control study that reported in 2015 (NCT00985517 [87]).

Patients in this study now received bilateral doses AAV2-NRTN to both SNc and putamen, with putaminal doses being four times the previous dose, and subjects being followed for between 15 and 24 months. However, again the study failed to hit its primary endpoint of change in defined OFF UPDRS part 3 motor scores. Notably, given the pathological data, disease durations for participants in this study were slightly shorter than before, at 7.8 years for the treated and 8.6 years for the sham cohort. As fluctuations are a marker of NS degeneration, this suggests that denervation of the striatum in these subjects was probably still profound. One of these subjects has come to postmortem subsequently (8 years after surgery), and this examination confirms that vector-mediated NRTN expression remains robust even at this time point, with correlated TH expression, but that the coverage of expressing tissue was again very limited in extent [122].

Despite its overt failure, post hoc analysis of this trial again suggested that certain subgroups, in particular those with shorter disease durations, might show some benefit, backing up the pathological conclusions. This latter point is a crucial insight and one that is being tackled in the inclusion criteria of an upcoming AAV2-GDNF trial being run through the Universities of Ohio and California.

### AAV2-GDNF

On this background, AAV2-GDNF is the last therapy still standing in the arena of GF GT for PD at present, and recent reports suggest some hope. Preclinical work suggests that GDNF may have more powerful neurotrophic effects than neurturin [123] [98], and successful transduction of putaminal medium spiny neurons has been shown in both rodent, primate, and aged primate models with rescue of dopaminergic projections [124–126].

Following on from these preclinical studies, a phase 1 trial of 13 patients with moderately severe PD was undertaken sponsored by the National Institute of Neurological Disorders and Stroke (NINDS) (NCT01621581) [88, 127]. AAV2-GDNF was delivered by iMRI-CED in 3 dose levels between 9 × 10^10^ and 9 × 10^11^ vg per patient, and the procedure was well tolerated. Gd-iMRI tracking of the infusions however showed that putaminal coverage (using 450 μl infusate per putamen) was again limited at only 26%, suggesting that, despite demonstrated improvements in ^18^F-DOPA PET signal, further optimization of delivery might be required in future studies. This conclusion was also reinforced by the variability in improvement in PET signal; although improvement was seen in 12 of 13 patients, the range of change in those that improved was wide (between 8 and 130% above baseline, median 54%), and the other patient experienced unilateral deterioration.

Despite these limitations, the NINDS study has provided proof of principle evidence, and a second study (NCT04167540) is now being planned. This new study is penned to start imminently, based out of the Universities of California and Ohio, and sponsored by the biotech company Brain Neurotherapy Bio, Inc. Its twin aims are to assess the utility of GF GT in a cohort of patients with earlier disease (less than 5 years) and also to optimize tissue coverage using iMRI and a novel neurosurgical approach. This uses a posterior stereotactic trajectory and a single infusion cannula that should facilitate better coverage of the irregular volume of the putamen—allowing a “shape-conforming” infusion [128]. The primary endpoint is safety over 5 years, with secondary endpoints of changes in motor and nonmotor scores, alongside DAT scan changes (18 months).

### GT with Growth Factors—Still a Viable Option?

Overall, these GF studies have been very valuable, in particular with respect to our improved understanding of the pathological human striatal environment in PD. The good safety and tolerability profiles of the AAV2 vectors that has emerged is also important, but the overarching conclusion is that using GF-mediated GT to rescue the degenerating human NS system in PD is unlikely to be a panacea for all patients.

Even so, the potential prize remains substantial, and if a clear “disease severity threshold” could be drawn, before which GT could be given with a good prospect of benefit, then this would still be an important achievement. PD is relatively easy to diagnose, and such a therapy given early (e.g., before 5 years) would have the potential to slow disease progression in this pathway across large numbers of patients, so ameliorating a large burden of disease that currently carries very significant impacts on patients, carers, and society.

Unfortunately such a threshold—a measure of responsiveness to GF therapy—is likely to vary considerably from patient to patient (perhaps up to 10 years in some patients) and may be difficult to assess accurately a priori. However, there is currently a good prospect that the DA replacement strategies (AAV2-AADC and LV-triple enzyme) will become important backup options in later stage patients, alongside the current mainstay of DBS. By comparison, the circuit modification approach seems to have faded as an option at present, but its ease of surgical administration to a small volume target means that it is still an attractive alternate approach if significant benefit can be demonstrated.

### Growth Factors Long Term

The prospect of treating PD patients early with GF GT is not only enticing but also carries risks. Short-term side effects of the GF treatments thus far have been minimal but the longer term risks are very unclear. NHP data has not so far produced clear evidence of long-term problems, but potential side effects, including aberrant sprouting of SN DA neurons, weight loss, and hyponatremia, have been highlighted in some preclinical work, as well as one of the early trials that delivered rGDNF into the cerebral ventricles [101, 129, 130].

For these reasons, some attention has switched to looking at regulatable vectors that could allow the GF stimulus to be switched off flexibly after transduction—perhaps even allowing an intermittent GF dosing regimen to be used over months and years. This sort of regulation would require additional gene regulatory sequences and might be difficult to achieve within the restrictive payload of AAVs. This issue might drive a move to use LVs for GF therapy in due course, as these vectors can facilitate regulatable expression in different cells systems including the delivery of GFs to the striatum [131]. However, there remains a good deal of focus on developing AAVs in novel ways [24], so regulatable AAV technology may yet emerge.

## Future of Gene Therapy for PD

GT therapy is still in its early days and is an emerging technology. Several new avenues suggest that the studies described above may only be the forerunners of a wider array of GT options for PD in the future.

### Combined Therapies

Although combining GF therapy with DA synthetic GT has been postulated, it is unlikely to be a useful strategy given that different disease time points require different therapeutic strategies. By the stage of disease at which DA synthetic treatment needs consideration, GF GT would be unlikely to work given the extent of NS degeneration; conversely, early treatment with GF GT ought to obviate the need for later GT to manage motor fluctuations.

A more likely scenario is that GF GT will be combined with, or replaced by, other GT approaches that either promote responsiveness to GFs or otherwise provide a beneficial disease-modifying effect. Although systemic (non-GT) treatments along these lines may well be feasible and easier, the GT approach may still retain some advantages. By “vectorizing” a treatment to certain neuronal populations (e.g., NS projection neurons or striatal MSNs), off-target effects might be minimized and a more potent disease-modifying effect obtained. Novel approaches along these lines might include manipulation of downstream pathways from NRTN/GDNF (e.g., overexpression of the GF receptor Nurr1), or direct targeting of culprit disease pathways, including α-synuclein toxicity [132, 133], and LRRK2 kinase overactivation [134]. Virally mediated gene inhibition (RNAi) or editing (CRISPR-Cas9) are two options here [135], but where PD is seen in the context of *GBA1* mutations, expression of the normally functioning wild-type gene may prove beneficial [136]. A New York-based biotech company is already pursuing this latter goal using intracisternal injection of an AAV9-based vector carrying *GBA1*, with phase 1 results likely to be published in the near future (NCT04127578 and [137]). Also on the brink of clinical testing is vectorized antibody therapy against α-synuclein, a technology that has been previously been developed for treating HIV infection [138, 139] and which is being pushed in the PD field by a collaboration between pharma companies Abbvie and Voyager. Again, an AAV vector is the proposed technology [140].

### Other Vectors

With the AAV2 and EIAV studies being generally well tolerated, there will be a good deal of temptation to stick with vectors known to be safe. Introduction of a novel vector would entail starting from scratch with safety studies for any evolving strategies, but such approaches may offer new potential advantages. In particular, AAV2 is known to bind to heparan sulfate proteoglycans (HSPGs), moieties that are abundant on the neuronal surface and contribute to vector uptake, but which also limit vector diffusion. Although this has allowed tight control over vector targeting, arguably this control has been *too* tight, contributing also to the limited putaminal distribution of vector that has led to failed trials. Other AAV serotypes, such as AAV9, can diffuse more broadly within neuronal tissue [15], while maintaining neuronal infectivity, and may well have achieved better putaminal distribution of vector payload had they been employed. Such vectors, and modifications of them, may yet come into their own for PD, in particular for strategies that aim to curtail the spreading pathology that characterizes the PD brain. Some of these vectors may also allow peripheral (or intrathecal) delivery (see below).

### *In Situ* Reprogramming

Although the strategy of asking putaminal cells to start making DA *in situ* is easily understood, another option being explored is a more fundamental reprogramming of endogenous putaminal cells, converting them directly into TH expressing neurons. These brand new DA neurons (perhaps converted from endogenous astrocytes) might be able not only to start producing DA, but also to sprout an axonal arbor and provide more efficient DA delivery to striatal cells via synapses. The technology for this, although more distant than standard gene therapy, is nevertheless on the horizon, with encouraging data emerging in animal models [141].

### Bypassing the Surgeon

Future nonsurgically delivered therapies may also yet displace these options, particularly if delivery methods are eased while target specificity is maintained, and any immunological issues are side-stepped. One option proposed is the use of peripherally delivered vector (e.g., via intravenous infusion), in combination with targeting to specific brain regions by local disruption of the blood–brain barrier (BBB) with focused ultrasound [142]. This could be an extremely attractive option for either advanced or early disease. Indeed, more anatomically diffuse disruption of BBB can be obtained even more easily pharmacologically [143] and might enable much more widespread CNS uptake of vector and, with this, more extensive disease modification.

## Summary of GTs in PD

We are currently at an exciting time in the arena of GT for the PD field. Several therapeutic options are emerging in parallel as potential treatments for this condition, but with the safety profile of the technology so far holding, this bodes well for an even wider array of GT options in the future. Time will tell which if any of the current and future candidate GT treatments emerges as useful and practical therapy and so ends up dominating the therapeutic arena for PD. It is also possible that GTs could be used synergistically with cell therapies [144].

## The Cell Therapy Approach to Treating PD—the Rationale

The main rationale for the use of cell therapies in PD has been to replace the lost A9 DA neurons of the SNc—the ones that are principally affected by the disease process in this condition. Other approaches have been tried which exploit less defined modes of action—e.g., Spheramine® was trialled as a cell therapy that was thought to work more through a trophic and paracrine action [145], in much the same way as the parthenogenetic stem cell therapy being trialled at the present time by ISCO [146]. Nevertheless, the vast majority of approaches have sought to replace the few hundred thousand DA neurons that are lost in PD, engrafting new cells in the striatum where DA normally works. These therapies therefore are not being used to rebuild the circuitry as originally lost (since the grafts are placed heterotopically), and nor are they being used to cure PD—as the underlying disease process carries on in the PD patient brain and may even involve the grafted cells themselves (see below). Rather, DA cell therapies are being used as a symptomatic therapy to better treat the nigrostriatal dopaminergic aspects of PD, albeit that a core component of the PD pathology is being repaired in the process. Although this approach is restricted in its ambition, it does have the potential (if it works) to make redundant almost everything we currently do for PD patients in the clinic, with drugs and surgery. This is because all current drug and surgical therapies essentially work on the dopaminergic aspects of PD, and the complications that these drugs produce arise through abnormal activation of this pathway (see above).

## The Types of Cell Used for Transplantation

There are a number of cell types that have been considered to be suitable for grafting as DA neuronal replacements (including for example, peripheral sympathetic neurons, adrenal medullary cells, and carotid body cells), only some of which actually form neurons of the type lost in PD. This is important as preclinically it has been shown that the A9 neurons are the optimal DA cells for effecting repair at this site [147], and other DA cell types work less well, if at all. Many of the other sources of cells that have been trialled either do not form neurons (e.g., adrenal medullary cells [148]), or do not form the DA neurons of the midbrain (e.g., parthenogenetic stem cell-derived neurons [149]). In this review, we will concentrate on the work that has been done with fetal ventral mesencephalic (VM) tissue (that contains the developing DA neurons of the midbrain) and stem cell (both ES and iPSC)-derived DA neurons. In terms of these latter cells, some comment needs to be made at this stage about their relative advantages and disadvantages.

Human fetal VM (hfVM) tissue is derived from elective termination of pregnancies and as such brings with it major ethical issues around abortion. Thus the use of such tissue is strictly regulated in all countries, but in some it is not allowed to be used at all for clinical purposes and/or research—although this can also change over time (e.g., the USA). In part, this is because of concerns that the adoption of such an approach may encourage women to have terminations, especially if they have family members affected by conditions that may use such tissue for therapeutic gain. In addition, religious objections are also key arguments raised around not allowing human fetal tissue from termination of pregnancies to be used, centering on questions of the potentiality of embryos and the sanctity of life.

However, in the UK for example, there is no evidence that using fetal tissue in transplant programs changes practice in terms of women deciding to have a termination of pregnancy or not. Furthermore, there are strict guidelines stipulating that those donating fetal tissue cannot say which programs of work they want that tissue to be used for, either clinically or for research. In addition to these ethical problems are the logistical ones that in order to have enough surviving DA neurons after grafting, tissue needs to collected from at least 3 fetuses per side of the brain grafted. This puts major constraints on having sufficient tissue to graft patients, given that hfVM tissue cannot be cryopreserved successfully and can only be stored in hibernation medium for short periods of time (a few days only) [150]. Thus, in our recent trial using this tissue in patients with PD (TransEuro), 87 planned transplant operations were cancelled because of insufficient tissue being available for grafting [151]. A final major problem with using this tissue is that the final grafted cell product cannot be standardized as each patient will receive their own unique tissue transplant, which also contains many cells of the type not needed, given only the minority of cells in the developing VM are of midbrain dopaminergic origin. However, there are advantages with using this tissue, including that the use of primary, non-manipulated tissue brings many regulatory and safety advantages, as well as greater confidence that one is replacing like with like—namely the DA cells of the midbrain that are lost in PD are being replaced with genuine human midbrain A9 neurons.

The use of human pluripotent stem cells gets around many of the above problems, especially logistical ones given these cells can be easily expanded and differentiated in culture and then cryopreserved [152, 153]. However, such stem cell therapies are still not without problems. Thus with human embryonic stem (ES) cells, ethical concerns remain around the fact that the cell line so derived can only be achieved through destruction of that embryo. Some have tried to circumvent this problem using single blastomeres, so in theory side-stepping the need for embryo destruction [154].

This problem is avoided to some extent with human iPSC lines, although not completely, as questions of potentiality still exist with any human stem cell (hPS) [155]. In addition, the country of origin of the human stem cell line also has implications, as lines derived from countries that have had cases of new variant CJD cannot currently be used easily in the USA. Given the size of the US market, this has major consequences on the commercial development of any product derived from such cells — the investment in and thus development of that product may be significantly limited. Finally, there are issues that have yet to be resolved about the genetic testing and variability seen in stem cell lines, and what this actually means for their safety clinically. Although many different types of genetic variability have been shown in a range of cell lines (e.g., hPS cells recurrently acquire and expand dominant negative P53 mutations) [156, 157], this also varies as a function of time in culture, and the significance of the variability from a safety perspective is still unknown. The position of the regulatory agencies on this aspect also remains to be defined.

All of these issues, coupled with the commercialization of any future stem cell-based therapies for PD, has meant that many teams are now seeking to derive and use their own hPS cell line, as this gives them greater control over what that line can be best employed to treat. This though can make it hard to extract exact information about the cell being developed for clinical trialling. However, reassuringly, most modern differentiation protocols mean that the major worry with these cells of teratoma development is a problem that is more theoretical than real. In addition, the regulatory agencies now all have clearer guidelines on what is needed to show that these cells are acceptable from a safety, biodistribution, and tumorigenicity perspective.

## Immunological Issues with Cell-Based Therapies

The use of cell-based therapies for treating PD brings with it issues of immunogenicity, as would be the case for any allotransplant. One difference for CNS disease is that the brain has a degree of immunological privilege, and the cells are also not especially immunogenic given their fetal origin or derivation. Nevertheless, rejection issues are still important and need to be considered, as they may have contributed to the lack of efficacy in the double blind placebo-controlled fetal transplant trials done in the last part of the twentieth century (reviewed in [158]).

The brain has long been known to have relative immunological privilege by virtue of having no professional antigen presenting cells within it, a limited lymphatic drainage system, and a blood–brain barrier (BBB). However, of late, it has been shown that the CNS is actively patrolled by the immune system and that a relatively well-developed G lymphatic system is also present, which means that antigens within the CNS can be delivered to the peripheral immune system and an immune response generated [159]. As such, cells placed into the CNS will likely evoke an immune reaction, especially as the grafted procedure itself will temporarily disrupt the BBB. Thus, unless the cells are autologous, some sort of immunosuppressive treatment will be needed at least for a time after implantation.

The extent of this immunosuppression will depend in part on the following factors:

- When the BBB seals up (which is thought to be within a few weeks for cell suspension grafts [160]).
- When dying cells and fragments from the transplant stop entering the peripheral immune system and thus being exposed to the peripheral immune system (which is probably in the first few weeks only after grafting [161]).
- The immunogenicity of the cells being grafted, both at the time of grafting and as they mature relative to the host.

For hVM tissue, this last issue has been looked at both *in vitro* [162] and *in vivo* [163, 164], with such work showing that the tissue can both express major histocompatibility (MHC) antigens and invoke an immune response. This has also been seen with hPSC-derived allografted tissue *in vitro,* as well as in NHP studies using matched and mismatched stem cell grafts [165]. In this latter study, it also shown that the rejection response could be abrogated with tacrolimus monotherapy [165]. All of this has led some groups to consider less immunogenic cells—either using an autologous approach [166], or using a universal cell line where the major MHC antigens have been engineered out of the cells [167]. Whether either of these approaches really offers advantages for treating PD remains unproven, as is the case that autologous iPSCs are really immunologically tolerated when grafted back into the donor host [168]. There are though two other issues that need to be considered with the use of autologous approaches:

1. The cost of making individualized cell therapies (which currently in the UK would be in the region of several million pounds per patient)
2. The risk of promoting any PD pathology in the grafted cells given they are patient derived (and thus harbor the same genetic risks for PD) and α- synuclein pathology has consistently been found in unrelated fetal DA cells 10 years after grafting [169]

In spite of these possible risks, the first report has been published of a single patient receiving an autologous iPSC derived DA cell transplant for their PD [170]. In this case, the patient had bilateral transplants of cells without immunosuppression and responded well symptomatically, although not on other more objective measures. This case, while showing that this approach was safe, used protocols for making the DA neurons that might not have been optimal and in addition there were other controversial aspects to how this transplant was done [171].

At the present time though, most groups have elected to use allogeneic cells and immunotherapy after grafting. This latter regime is typically of the type normally used in other organ transplants and which to date has been used with fetal tissue trials in patients with PD without much in the way of complications [151]. As to exactly what this regime will look like in future stem cell trials for PD, and for how long it will be given after grafting, is still to be finalized.

## What Has Been Shown with Cell-Based Therapies to Date?

The initial trials in the early to late 1980s predominantly used adrenal medullary tissue for which the preclinical evidence was limited (reviewed in Barker and Dunnett 1991). The conclusion of the clinical trial work using this tissue, which was undertaken extensively across many centers especially in the USA, was that these transplants offered no real benefit and were associated with side effects and poor survival [172, 173]. Therefore they were abandoned, especially as more encouraging data was beginning to emerge with the use of hfVM allografts.

The move to trial hfVM in the clinic came on the back of robust reproducible preclinical findings in neurotoxic animal models of PD that had been carried out in many different laboratories around the world. These studies showed that allografts of the developing VM into rats lesioned unilaterally with 6-hydroxy-dopamine (6-OHDA) could survive, make and receive connections from the host brain, release DA, and restore behaviors to normal in these animals (reviewed in [174]). This was also shown in NHP, most notably marmosets [175], and it was on this background that patients were first grafted in the late 1980s and thereafter till the end of the century.

Although a number of groups took on this early work, the best studied cohort was a series of PD patients recruited to an iterative open-label study in Lund Sweden, led by Olle Lindvall and Anders Björklund, which also included two MPTP patients from California [176]. All this work involved grafting patients with relatively advanced PD with hfVM tissue, and following them over extended periods of time both clinically and with PET imaging of the DA system. This study, along with other open-label studies showed that transplants of hfVM could:

- survive long term in the human PD brain (e.g., [177]).
- have long-term clinical benefits (e.g., [178]).
- release DA in a physiological way when the graft was pharmacologically stimulated (e.g., [179]) with re-activation of motor cortical areas after grafting [180].
- improve quality of life measures not just motor features (e.g., [181]).
- work as well when placed striatally as when placed in the striatum as well as the nigra [182].
- not be guaranteed to work in every patient (e.g., [183]).
- acquire the pathology of PD after several years of engraftment (e.g., [184]).

These early studies led to more formal studies being undertaken in the USA in the early 1990s once federal funding became available under the Clinton administration for work using human fetal tissue. These two NIH funded double blind placebo-controlled studies reported in 2001 and 2003 and failed to show clinical benefits that satisfied the trials primary end points [163, 185]. In addition, both trials reported for the first time severe and disabling graft-induced dyskinesias (GIDs) some of which required further neurosurgical intervention [186], and more recently, one patient was shown to have a transplant that did not work clinically despite recovery of striatal DA innervation at postmortem and on DA imaging [187]. As a result, the adoption of this approach for treating patients with PD fell out of favor, especially in light of the side effects and lack of efficacy compared with the results that were appearing at this time with DBS [188]. This in effect led to a moratorium on this therapeutic strategy and debates around whether cell therapies had any future in PD.

As part of these discussions, a review of the data from all these trials was undertaken [158, 189], and a number of conclusions were drawn, suggesting that this approach may still have merit, especially given that protocols for making human stem cell-derived DA cells of an authentic midbrain type were starting to emerge. These conclusions were that:

- Those patients that seem to have the highest chance of benefitting from hfVM grafts tended to be younger with less advanced disease.
- Patients with extensive striatal DA loss that extended into the ventral stratum did less well [190].
- Patients who developed GIDs had reported significant levodopa-induced dyskinesias (LIDs) pre-grafting, but also that the development of GIDs may relate either to a poor distribution of DA cells across the striatum [191], or to the grafting of large numbers of 5HT neurons relative to the DA cells in the transplant [192]—all of which could be avoided using better preparation and delivery approaches.
- The use of no immunosuppression in the first NIH study was associated with low numbers of surviving DA cells at postmortem, although there may be other reasons for this relating to the amount of hfVM tissue grafted. In addition, in the second NIH-funded study, there was a distinct change in the direction of the clinical response of grafted patients when their ciclosporin A was stopped 6 months after surgery. Although the reasons for this remain unknown, it could relate to a partial graft rejection on stopping the ciclosporin A.
- Clinical improvements could take many years to appear and thus using primary end points relatively soon after grafting may miss the maximal benefit of the grafts.

As a result of this, a new trial funded by the EU (TransEuro) was set up that tried to address many of these factors in its design. This trial, which grafted its first patient in 2015 and its last in 2018, will report next year at the earliest, as the primary end point is the change in the UPDRS part 3 score in the defined OFF stage, 3 years after the second transplant. The lessons learnt from this trial have recently been published with the hope that this will help in the design of the next round of stem cell-derived DA cell transplant trials for PD [193]. In addition, this article also highlighted why this therapeutic approach, using this cell source, has no future from a simple logistical perspective, but may be adapted if a stem cell source of DA cells can be identified.

In this last respect, in 2011 and 2012, two seminal papers from the groups of Studer and Parmar published protocols showing that hESC could be converted into authentic DA neurons of the midbrain type. Theoretically, these could therefore be used for treating patients with PD in much the same way as hfVM tissue [194, 195]. Subsequently, it has been shown that this approach offers major potential therapeutic promise in treating PD, by virtue of the fact that these DA neurons can:

- Be produced reproducibly, with a midbrain DA phenotype, using different starting cell lines [196].
- Be manufactured in the numbers needed for clinical translation in a GMP compatible fashion [197].
- Be shown to have equivalence to human fVM grafts in terms of their functional effects in animal models of PD [198].
- Connect and establish connections appropriately with the host brain [199, 200].

In addition, grafts of these cells do not form tumors or contain large numbers of 5HT neurons [201]. As a result, several academic groups, often in conjunction with pharma or small Biotech companies, around the world are now moving this technology towards clinical trials including in Europe, the USA, and Japan, with the first of these having started with an iPSC approach in Kyoto in Japan. This work has been led by Jun Takahashi, based on his preclinical work in parkinsonian NHPs, where he showed long-term survival and efficacy of human iPSC-derived DA cells [202].

All these groups have over the last 5–6 years worked together as a consortium (GFORCE-PD [193]) to try and ensure that their respective clinical trials can be better coordinated, and also to facilitate the exchange of data around preclinical developments of these stem cell-derived DA cell products [203]— an approach which we encourage others working on similar experimental therapies to adopt. These trials, which were due to start in the USA in 2020 and in Europe in 2021 prior to the corona virus pandemic, have attracted commercial interest from major companies as the potential for this therapy to transform the natural history of treated PD at an affordable cost has now been perceived as realistic by many working in this field.

## What Are the Challenges Moving Forward with This Approach and Where Will It End?

The field of stem cell-based therapies for PD sits at an important stage in its development, since it is about to enter clinical trials where the effectiveness and utility of this therapy will be revealed, along with its competitiveness—both therapeutically and commercially. If these early trials prove successful, then one can imagine that this therapy will be moved to be trialled in earlier stage patients as there is a logic in doing this especially in younger onset cases of PD. Ultimately this therapy has the capacity to transform how we treat PD by obviating the need for many if not all of the therapies currently given in the clinic to patients with PD for their motor problems. Although this remains a long way off, the commercial investment in this area does mean that this field is likely to move faster through phase 2/3 trials than we might imagine.

As to where next, there is great interest in using the technologies we have described in this review to directly reprogram cells *in situ* without the need to graft cells into the brain (see above). In other words, can we convert resident glia into DA neurons and by so doing avoid the practical, ethical, and immunological issues that play a role in any cell transplant grafted into the brain? This is now being explored in animal models, although to date the results have been modest at best with little evidence that sufficient DA cells can be generated of the type needed to reverse the deficits seen in PD [141, 204]. Nevertheless, this whole area is one that is changing every year as our ability to reprogram cells becomes ever better and with this the prospect of repairing the PD brain from within.

## Electronic Supplementary Material

ESM 1 (PDF 1224 kb)

ESM 2 (PDF 1224 kb)
